# On the Application of Laser Vibrometry to Perform Structural Health Monitoring in Non-Stationary Conditions of a Hydropower Dam

**DOI:** 10.3390/s19173811

**Published:** 2019-09-03

**Authors:** Mateja Klun, Dejan Zupan, Jože Lopatič, Andrej Kryžanowski

**Affiliations:** Faculty of Civil and Geodetic Engineering, University of Ljubljana, Jamova 2, 1000 Ljubljana, Slovenia

**Keywords:** vibration, laser doppler vibrometry, concrete dam, accelerometers, dam monitoring

## Abstract

This paper presents the first application of the Laser Doppler Vibrometer (LDV) in non-stationary conditions within a hydropower plant powerhouse. The aim of this research is to develop a methodology to include non-contact vibration monitoring as part of structural health monitoring of concrete dams. We have performed in-situ structural vibration measurements on the run-of-the-river Brežice dam in Slovenia during the start-up tests and regular operation. In recent decades, the rapid development of laser measurement technology has provided powerful methods for a variety of measuring tasks. Despite these recent developments, the use of lasers for measuring has been limited to sites provided with stationary conditions. This paper explains the elimination of pseudo-vibration and measurement noise inherent in the non-stationary conditions of the site. Upon removal of the noise, fatigue of the different structural elements of the powerhouse could be identified if significant changes over time are observed in the eigenfrequencies. The use of laser technology is to complement the regular monitoring activities on large dams, since observation and analysis of integrity parameters provide indispensable information for decision making and maintaining good structural health of ageing dams.

## 1. Introduction

Every year about 200 new large dams of different types are built worldwide; however, the intensity of dam construction was even greater in the past, therefore the majority of the dams that will operate in the 21st century already exist [1]. Consequently, ageing of dams, preservation of their functionality, and preservation of structural health are becoming the main challenges of the dam engineering community [2]. Hydroelectricity has a significant role, while hydropower turbines also provide ancillary services for the electrical network: maintenance of the system frequency (due to fast and automatic response), fast reserve, reactive power series, and black-start capabilities [3]. With the inclusion of more renewable power sources in the grid, which are known to be less stable, the role of water turbines has increased. Most run-of-the-river hydropower projects were designed to provide for the base load, but nowadays they provide for variable and peak regimes as well [4]. Hydropower plants still remain the most flexible element in the grid to perform continuous regulation. In the past only a few units were sacrificed to be highly flexible in operation, while nowadays due to the changed conditions practically all hydro units in operation on the grid are continuously regulated and daily operate in transient and unsteady modes [5]. This sacrifice is already recognized in more frequent down times of turbines [6,7]. Furthermore, Goyal et al. concluded from laboratory testing that each start and stop procedure causes a fatigue damage equal to 15–20 h of regular operation [8]. Operation in off-design operation points not only causes fatigue to the turbine, the disturbances and stress fluctuations transmit further into the bearing structure, to the first and second stage concretes surrounding the turbine, penstock, spiral, and further [9]. The cracks in the foundations found at some powerhouses are suspected to be linked with the new patterns of operation [10]. In extreme cases the extent of damage called for vast rehabilitation works [11]. With the focus on the hydropower dams, turbine operation is a load that occurs daily and its effect accumulates over decades. This low amplitude, high frequency cyclic loading has, in comparison with other dynamic loads, a minor impact, however, the accumulation of damage and its contribution to fatigue of the bearing structure has not yet been fully considered and investigated. Nevertheless, in order to successfully manage and maintain the ageing inventory of dams this phenomena should also be investigated.

The accumulation of damage in a structure causes changes in the dynamic properties which can be detected with vibration monitoring. In order to be able to perform structural diagnostics, a system has to be well investigated. Due to the size of the object under investigation, it is impossible to create series of specimens, introduce different types of damage, and measure the response. We can only depend on the measurements during operation while over the years of monitoring we will be able to gain new knowledge on the ageing and behaviour of the system. In the initial stages it is important to define the monitoring programme and decide which technology is used for the task [12]. Accelerometers are probably the most commonly used vibration monitoring sensors [13]. However, laser vibrometers are an interesting alternative to the traditionally used contact measurements. Cristalli et al. (2006) performed a comparative study between laser Doppler vibrometers and accelerometers to be used in an on-line testing to detect the fault of motors at the end of the production line [14]. The study concluded that both technologies have their advantages and disadvantages. The vibrometer velocity signal faces troubles of identifying higher modes because of the noise threshold and the vibration of the laser head, while accelerometer measurements are affected by the electromagnetic effect and with installation constraints, which in some cases prevented feature extraction for fault diagnosis. The vibrometer velocity signal proved to be the most appropriate for fault diagnostic at the on-line quality control of motors at the end of the production line.

In this paper we present a methodology on how the laser Doppler vibrometer can be used inside a hydropower plant to measure vibration, while the instrument is excited as well. First, we present the basic working principle, the experimental environment, and the procedure of error extraction. The test measurements are performed at the Brežice dam on unit 1 during regular operation, with the placement of the laser a few meters away from the measured point, where the instrument is excited with the operation as well. In this paper we present the procedure of the measurement, mathematical operations, and the final result compared with the control measurements. We conclude with the recommendation on the use of the procedure, on the work that has been already concluded, and the aims for future work where non-contact measurements are included in the regular monitoring activities on the dam.

## 2. Methods

The laser technology was invented shortly after 1960. A few years later the laser Doppler anemometry (LDA) as a novel technology to measure fluid velocity was introduced, and finally in the late 1970s the laser Doppler vibrometry (LDV) was invented [15,16]. Both technologies advanced since then and are nowadays widely used in different areas of applications, e.g., vibration measurements of the human middle ear, non-intrusive diagnostic of fresco paintings, the performance of underplatform friction dampers for turbine blades [17,18,19]. In a review paper An International Review of Laser Doppler Vibrometry, the leading authors in this field present a detailed review of the chronological and technological advancement in the field [20].

The working principle of LDV is the Doppler effect in the laser light, while there is a direct correlation between the Doppler shifts $f_{D}$ of the backscattered light from the moving surface and the surface velocity *v*:

(1) $$f_{D}=\frac{2v}{\lambda }$$

where λ of HeNe laser light equals 633 nm [21]. The challenge of on-site measurements in comparison to laboratory measurements is the noise of the environment. In laboratory we are able to control ambient conditions, while on site we have limited options; basically the conditions are given and we have to adapt to them [22]. Measurements using LDV are limited to surfaces that provide sufficient amount of reflected light. The majority of the surfaces in civil engineering is at least to some extent optically rough with respect to the laser light wavelength. When laser light is reflected from a rough surface coherent waves of the incident laser beam are dephased and speckles are formed [23]. The measurement is possible when a speckle has sufficient intensity to be recognized on the photo detector. The amount of the backscattered light is also affected by atmospheric conditions (humidity, temperature), laser beam propagation, focus, and alignment [24,25,26]. The laser speckle is classified as a fundamental measuring uncertainty, while the laser signal can be a carrier of surface motion information unrelated to normal surface motion, known as pseudo-vibration [27]. An in-depth analysis of the origin of speckle noise is presented by Rothberg (2006) [28]. The use of a retro-reflective tape helps to increase the intensity of the light in the backscatter. The retro-reflective tape is also optically rough on the scale of the laser light; however, it is designed to concentrate the scattered light up a narrow cone which provides for a brighter concentrated speckle. The use of the retro-reflective tape increases the intensity of the light in the backscatter, however, it does not guarantee a signal clear of drop-outs, while it provides for a wider range of where acceptable levels of the signal can be obtained [29]. The maximum stand-off distance is limited with the product limitations; however, the actual range is usually less than specified, while the conditions are never completely optimal. The optimal stand-off distances are located within the maximum range at points of visibility maxima. In practice, the search for visibility maximum is in most cases unnecessary, while the devices are sensitive enough to make the measurement even in the lower visibility spectrum.

### Challenge for Non-Contact Vibration Monitoring during Full Operation of the Powerhouse

**Figure 1:**
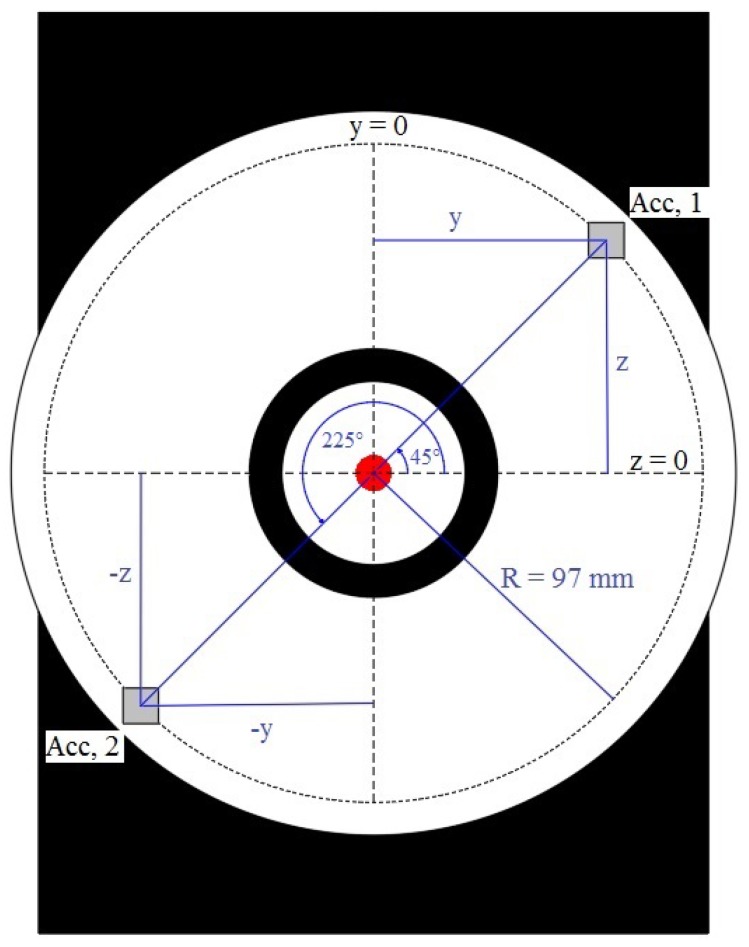
Installation of the accelerometers on the front mask of a vibrometer.

Vibrometer measurements are relative; if the device moves during the measurement, the output represents the sum of the vibration of the measured surface and the error caused by the movement of the device in the direction of the laser beam. With measurements inside the powerhouse during regular operation the tripod with the vibrometer is placed on an active standpoint, which is subjected to some sort of excitation as well. In some cases the standing point moves the same as the surface under observation. The first aim in the placement was to position the tripod on a structurally different member and in order to maximize the backscatter every experimental point is equipped with a reflective tile. To combat the issue of pseudo-vibration caused with the movement of the vibrometer, additional measurements to determine its movement need to be done. Halkon and Rothberg proved with laboratory and some real-world measurements that with placement of only 2 additional uni-axial accelerometers on the housing of the vibrometer the error caused by the movement of the device in the direction of the laser beam can be extracted [30]. The mathematical procedure and laboratory measurements where the procedure was validated are in detail described in the aforementioned paper [30]. Here, we will focus on the application in the powerhouse and the issues concerning measurements out of laboratory conditions. The placement of the corrective accelerometers has to be precise, and in order to smooth the error caused by the rigid body tilt of the vibrometer, accelerometers have to be placed on one of the diagonals and perfectly symmetrically to the origin of the laser beam, i.e., mirrored where the y,z location components should be $\left(y_{1},z_{1}\right)=-\left(y_{2},z_{2}\right)$ and also $y_{1}=z_{1}$ and $y_{2}=z_{2}$, where y=0 and z=0 represent the origin of the laser beam (see Figure 1). The precise installation is very important; a 1 mm imperfection in the position causes an error of $\frac{\pi }{360}$ mm/s and an angle misalignment causes a 1% error per degree of misalignment [30].

## 3. The Experiment

A robust methodology should enable measurements during various operating regimes, while the instrument is positioned on an active standing point. The scheme of the measurement set-up includes the following:

- Laser Doppler Vibrometer
- 2 uni-axial accelerometers
- DAQ box for simultaneous data acquisition
- Portable computer

**Figure 2:**
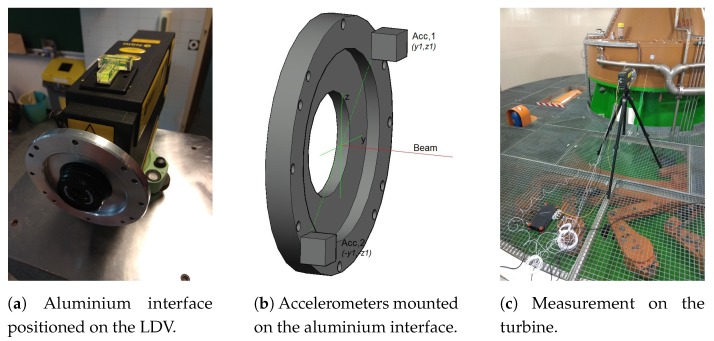
The interface mounting, scheme of the accelerometers positioning, and the measurement on the turbine in Brežice HPP.

As we were performing test measurements an additional accelerometer was used for control measurements. The 3rd accelerometer was mounted on the surface directly where the laser beam illuminated the measured surface. To ensure proper placements of the accelerometers on the vibrometer, we developed an aluminium interface to be mounted on the LDV (see Figure 2a). The aluminium interface has a design tolerance of 0.02 mm and precisely rests on the frontal face of the vibrometer. Exact positioning was done using a total station and geodetic measuring, and fixed with 3 bolts. The circular interface is a 10 mm thick aluminium plate with 12 sensory cavities on a 97 mm circumference with origin in the centre of the lens (see Figure 2b). The primary orientation of the vertical axis is through the centre of the lens, other sensory points are graduated with a 45${}^{\circ }$ and 60${}^{\circ }$ phase step enabling variable positioning of accelerometers on the interface. The coupling of the accelerometers with the interface is with UNC bolts compatible with the coaxial connector on the accelerometers. In our experiment 2 sensory cavities have been used until now; where 2 accelerometers were placed at 45${}^{\circ }$ and 225${}^{\circ }$ positions (see Figure 1).

On-site test measurements were performed on a turbine housing at Brežice HPP during regular operation. The experimental set-up is presented on Figure 2c. The vibrometer was placed on a tripod next to the turbine housing where the standing point was subjected to the excitation of the turbine operation. Polytec PDV-100 was equipped with 2 Dytran, A series, piezoelectric, uni-axial accelerometers, an additional accelerometer with the same specifications was mounted on the turbine housing to perform control measurements. Simultaneous data acquisition was done using the DEWESoft Sirius data acquisition box that enabled simultaneous acquisition of up to 8 sensors. Data acquisition was done in the time domain, and since we were interested in the velocity signal, all accelerometer signals were directly integrated, using DEWESoft software, that enables direct integration and definition of the acquisition parameters [31]. Integration was done following the middle Riemann sum rule. Sampling frequency was high, i.e., 20 kHz, and the low-pass filter with the cut-off frequency at 1 kHz was applied. Considering the Nyquist criteria a lower sampling rate would be sufficient, we decided to oversample and filter high frequencies in order to reduce the broadband noise floor of the system. Signal post-processing of velocity channels was done using MATLAB software [32]. To determine the phase lag between the vibrometer and the accelerometers signals we used the cross-correlation function [33]:

(2) $$R_{xy}\left(\tau \right)=\frac{1}{T}\int_{0}^{\infty }x\left(t\right)y\left(t+\tau \right)dt$$

**Figure 3:**
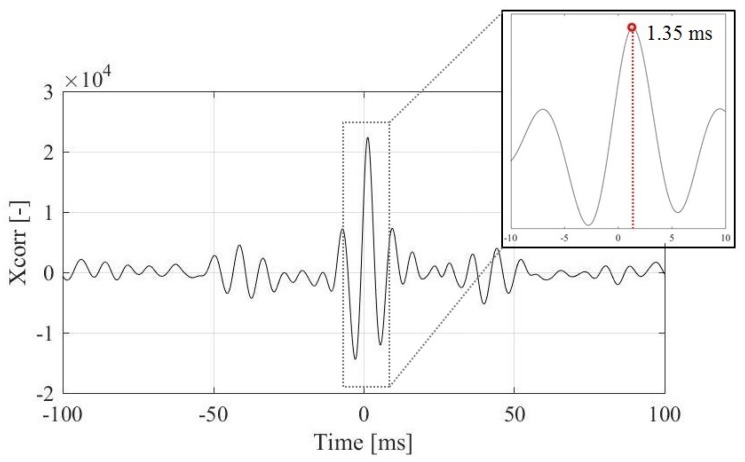
Cross-correlation value of Laser Doppler Vibrometer (LDV) and accelerometer signals. Local maxima at 1.35 ms indicate the accelerometer signal lags behind the LDV signal.

Cross-correlation function $R_{xy}\left(\tau \right)$ can be used as a measure of similarity between two discrete signals where τ represents the time delay and T the size of the data segment. Phase delay can occur even with a simultaneous data acquisition, e.g., when different technologies are used, filters are applied, cable lengths differ. A rough estimate of a phase delay due to the variable cable length is that 1 m of cable adds approximately $5\times 10^{-9}$ s (an estimate when the speed of light is assumed to be c = $3\times 10^{8}$ m/s) of a delay. Integration turns the phase for π/2, meaning that the integrated velocity signal will lag behind the acceleration signal. The maximum value of the cross-correlation function is in position where the signal similarity is the strongest, hence the indicator of the phase delay between the signals. The phase delay between the LDV and the accelerometer channels is 1.35 ms, where accelerometers lag behind the LDV signal, the output of the function is plotted on Figure 3, where we can observe local maxima at 1.35 ms indicating the accelerometer signal lags behind the LDV signal.

**Figure 4:**
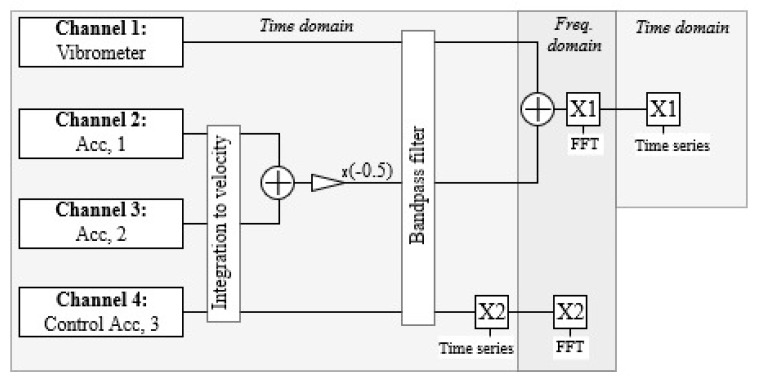
Scheme of mathematical operations on the output of sensor channels. Channel 1 represents the raw vibrometer velocity signal. Channels 2 and 3 represent the integrated velocity signal from accelerometers mounted on the vibrometer, while Channel 4 represents the control measurement of an accelerometer mounted directly on the turbine housing where the vibrometer light illuminates the surface.

Figure 4 represents the scheme of 4 channels during the measurements on the turbine after the alignment and integration of accelerometer signals. The first step in data manipulation is averaging of Channels 2 and 3, to omit the error caused with the tilt of the rigid body. Signals are then further transformed in the frequency domain using the Fast Fourier Transformation (FFT), signals are de-trended (linear trend is assumed), and the DC component removed. The frequency content of interest lies within 1–300 Hz. Using the digital band-pass elliptic IIR filter we attenuated frequencies below the lower edge and upper edge passband frequencies at 1 Hz and 300 Hz, respectively. The passband parameter is defined at 1 dB, while the lower passtop parameters at 80 dB. The use of the elliptic filter minimized the number of poles and the application of zero-phase digital filter prevented phase distortion of the filtered signal, while by processing the signal in both the forward and reverse directions, the phase remains unchanged. All signals were filtered including the control signal. Furthermore, the averaged signal from Channels 2 and 3 was deducted from the original vibrometer signal. Using reverse FFT the corrected time series were constructed. The result presents a corrected velocity signal of the turbine.

### 3.1. The Measurement Noise

**Figure 5:**
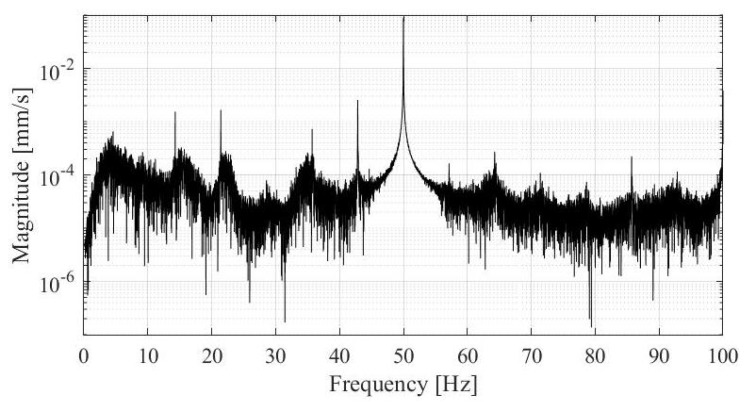
Noise at 50 Hz in the accelerometer output (output of channel 1).

The piezoelectric accelerometers used in this study were chosen for their small footprint and light mass for easy and reliable attachment. The accelerometer on the turbine was mounted with a magnet while the accelerometers on the interface were mounted with bolts. During turbine measurements we noticed a parasitic frequency at 50 Hz present in the output of the accelerometers. The parasitic frequency was more evident on the accelerometers mounted on the vibrometer than on the accelerometer placed directly on the turbine housing. The first suspicion was of course electrical noise and the effect of the magnetic field that develops in the powerhouse while the generators are operating, since it is known that accelerometers can be susceptible to the magnetic field excitation [14]. The output with the parasitic frequency is presented on Figure 5. As we can see the parasitic output dominates the measurements output.

The parasitic frequency is present only in the accelerometer output, while the vibrometer output at 50 Hz was only white noise. To confirm that the magnetic field causes the parasitic frequency content, we performed a detailed investigation in a laboratory. All components were tested for magnetic properties and exposed to the influence of a strong electromagnetic coil, designed for medical purposes. The coil induces a strong EM field with a frequency of 50 Hz. During the test at first the output of one accelerometer was monitored while the accelerometer was positioned on different surfaces (metallic, fabric, concrete) and on different distances from the magnetic coil. The electronic noise at 50 Hz appeared in the measurement as soon as the electromagnetic coil was turned on. The closer the mounting of the accelerometer the higher was spike in the frequency output. The accelerometer itself is mechanically moved by the EM field, the movement is so strong that it can be felt if we hold the accelerometer in our hand. The intensity of the movement reduces if the magnet used for mounting is removed, however the reduction is minimal.

**Figure 6:**
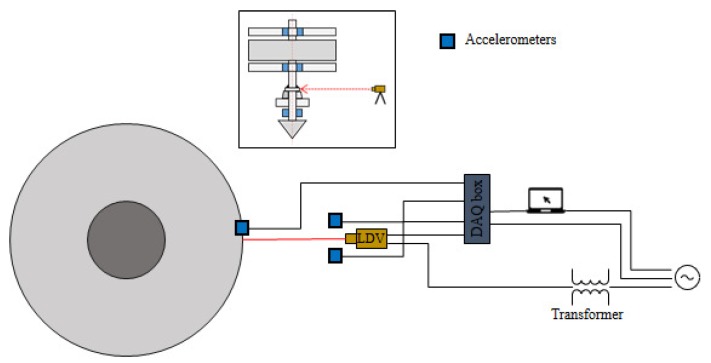
Layout of the instruments during vibration measurements, where the vibrometer is plugged into the isolation transformer. Vibrations are measured on the housing of the main bearing.

The Teflon covered cable is also a little bit magnetic: the magnetism is barely detectable, however, on the long length of the cable (15 m) the contribution is substantial. The effect can be minimised if the cable is always fully unrolled, while the rolled cable behaves like an electromagnetic coil itself. Furthermore, the vibrometer’s sensitivity to the EM effect was tested. Turning on the EM coil did not affect the vibrometer’s output. In the second step the accelerometers were added in the scheme. When the whole scheme was in operation, a parasitic frequency at 50 Hz appeared in the accelerometer output even before the EM coil was on; the effect barely rises above the white noise threshold. We added an isolation transformer in the scheme to introduce galvanic isolation in the system (see Figure 6). A galvanic isolation is used also to suppress electrical noise and isolate the plugged equipment from the original power-source. The transmission of the AC component is blocked while DC components are allowed to pass. This prevents the formation of a ground loop between two circuits (devices). The ground loop that forms between LDV-Sirius DAQ box-computer and affects the accelerometer readings was successfully suppressed with the introduction of the insulation. Based on the laboratory test we can conclude that the detected parasitic frequency consisted of the effect of the magnetic field and the ground loop. The latter is successfully eliminated with the introduction of the isolation transformer. By applying a digital band-stop filter, the parasitic frequency is completely eliminated. We use the band-stop IIR Elliptic filter with attenuation bandwidth from 49 to 51 Hz and 1 Hz transition band on the lower and upper edge frequency, pass-stop parameter 80 dB and peak pass-band ripple limited to 1 dB. The 50 Hz parasitic frequency is not near any system eigenfrequencies therefore filtering can be applied. With the presence of the magnetic field during the regular operation, the power plant environment once more proved to be a challenging environment for the measurements.

### 3.2. The Measurement of the Turbine during Regular Operation

**Figure 7:**
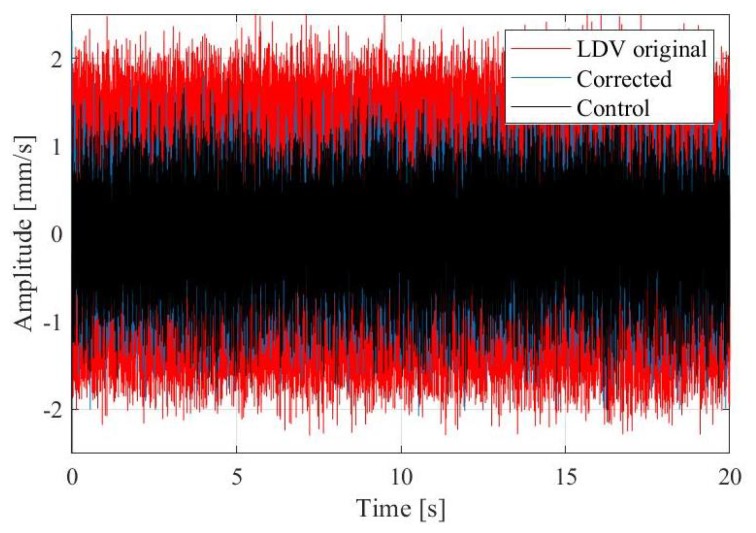
Comparison of the time series of the original vibrometer signal (red), control signal from the accelerometer (black), and the corrected vibrometer signal (blue) after the mathematical procedure is applied.

**Figure 8:**
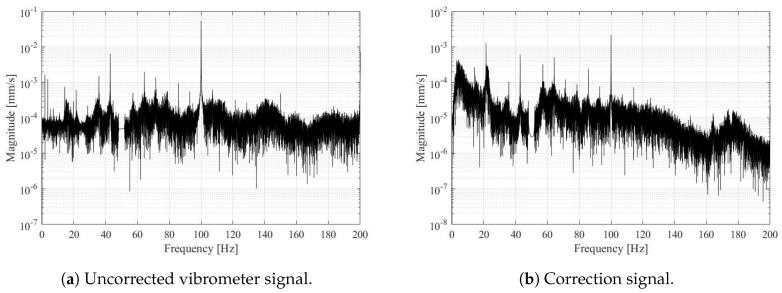
Frequency spectrum of the uncorrected vibrometer signal (**a**) and the correction signal from accelerometers 2 and 3 (**b**).

**Figure 9:**
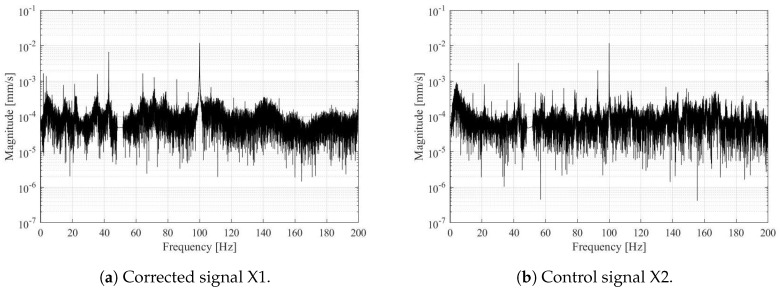
Corrected output signal (**a**) and the control signal that has been filtered and transformed in frequency spectrum (**b**).

In Brežice HPP station there are 3 vertical, double regulated Kaplan turbines installed. Our test measurements were performed on unit 1 (see Figure 2c). The vibration is captured on the turbine housing on the location of the main turbine bearing. The units rotate at 107.14 revolutions per minute. Including the stator each unit weights over 190 t and operates under 166 m^3^/s of rated discharge, the runner with 4 blades weights 35 t and the rotating part of the generator 96.3 t. As mentioned the vibrometer is placed a few metres away from the experimental point on an active ground, excited with the turbine operation. The raw vibrometer velocity output contains summation of the turbine vibration and error due to the movement of the standing point. Due to this additional movement the measured amplitudes are larger than the true vibration of the machine. Figure 7 presents the original vibrometer time series, the control accelerometer output, and the final, corrected velocity signal. Amplitudes of the raw vibrometer signal are larger than the control signal indicates, while after the corrective procedure the output amplitudes are in the range of the control amplitudes. The raw amplitudes exceed control measurements amplitudes by 80% or even more, the amplitude correction is therefore substantial. The correction is further discussed in the frequency plots on Figure 8 and Figure 9.

Figure 8a presents frequency spectrum of uncorrected, filtered vibrometer signal. We used the same filters on the LDV and accelerometer signals, e.g., also the band-stop filter. The correction signal is presented on Figure 8b. We can notice that the behaviour of the standing point is similar than the point under observation, the dominant frequencies are observed at 100 Hz, 42.9 Hz, 14.3 Hz, and 11.2 Hz. The highest magnitude is at 100 Hz with magnitude approx. $2\times 10^{-3}$ mm, the velocity magnitudes of the correction signal are at least one order of magnitude smaller than the vibration of the observed structure. The corrective frequency peaks also coincide with the structural frequency peaks.

Figure 9a presents vibrometer signal after the correction is applied, while in Figure 9b we present the control signal from accelerometer 3. Frequency peaks on both figures coincide, in Table 1 we summarize the most prominent frequencies in the power-spectrum in the range from 1–100 Hz, they are listed in the decreasing order in the spectrum and not by the order of magnitude. The three frequencies with the most energy during this measurement were 21.4 Hz, 42.9 Hz, and 100 Hz. The lowest peak in the frequency spectrum is at 1.78 Hz, this peak is a reflection of the rotational speed of the runner. Turbines rotate with 107.14 revolutions per minute. This is represented with the first peak in the frequency spectrum at 1.78 Hz. We can notice higher harmonics as well; the second peak at 3.56 Hz for example. The runner has 4 blades, the blade passing frequency at 7.15 Hz is recognized in the frequency spectrum as well. The higher harmonics are visible up to 200 Hz however, the 100 Hz, 150 Hz and 200 Hz peaks are not considered to be structural. In the accelerometer output they are considered as higher harmonics of the noise.

With the corrective mathematical procedure where the main signal manipulation is done in the frequency domain we were able to improve signal similarity. We used the value of normalized cross-correlation to be the measure of signal similarity, where the function from Equation (2) is normalized by the multiplication of the standard deviations $\sigma_{x}\sigma_{y}$.

(3) $$R_{norm}\left(\tau \right)=\frac{\frac{1}{T}\int_{0}^{\infty }x\left(t\right)y\left(t+\tau \right)dt}{\sqrt{\sigma_{x}\sigma_{y}}}$$

Normalized cross-correlation is a simple method to determine signal similarity and can range from values –1 to +1, where –1 means we are comparing two mirrored signals and +1 that we are comparing two exactly the same signals. The starting signal similarity ranged from values from 0.4 to 0.8 and after we applied the mathematical procedure the value was 0.9, which is a substantial improvement. However, at this stage we do not recommend for this technology to be used for blast loadings but rather to be used to measure during regular loading regimes. Blast loading scenarios have not jet been tested enough, the tripod on the stating point can move permanently, either pivot or translate. This can result in false or loss of data.

## 4. Discussion and Conclusions

The dam community is facing the challenge of ageing dam inventory, built at a time of different safety standards and economic regimes; many of these dams are now already extending the designed exploitation period. Inclusion of integrity parameters in the system of structural health monitoring of dams is going to be one of the necessary extensions of current monitoring activities. This study case evaluates the applicability and options for non-contact vibration monitoring. The existing monitoring system in Slovenia requires that seismic monitoring is established on all large dams. In our opinion a way forward is an extension of this system, where with the installed accelerometers for seismic monitoring the dam is continuously monitored under ambient condition and where local seismic events are recorded as well, providing useful information on system identification together with periodic non-contact measurements. The methodology is tested on the Brežice dam, and since there are 4 other dams on the lower Sava River which are structurally similar to this one, the methodology can be further directly transferred to those dams and in the future on other run-of-the river dams as well.

An extensive effort was devoted to extend the applicability of Laser Doppler vibrometry. A hydropower powerhouse is a demanding environment for relative non-contact measurements. The placement of the instrument inside the structure under observation results in an error caused by the instrument movement. The measurements on the turbine revealed that accelerometers are sensitive to magnetic field excitation. Additionally, electronic systems sometimes introduce a low level of instrument noise. Transverse motion and base bending of accelerometers and accelerometer cable noise have been recognized as a source of error. The issue was addressed with the introduction of an isolation transformer and a band-stop filter. Preparation of the measurement is crucial. It is important that we are familiar with ambient conditions and noise. When measuring on site we will most likely not be able to eliminate all noise sources however, we should be able to adapt to the conditions. The effect of ambient noise can be minimized by following a few simple rules:

- we recommend to always use reflective tape (unless we have a clear reason why not to use it);
- whenever possible the standing point should be on a structurally different member than the surface under observation;
- the standing point should be on a structurally more rigid member than the point under observation;
- when measurements are done in strong sunlight the visor of the instrument must be shaded;
- methods to enhance signal-to-noise ratio should be applied together with an adequate anti-aliasing method with a proper measurement resolution.

At this point we are able to use LDV in the powerhouse during the normal operation. The error caused with the movement of the device could be isolated and vibration of the turbine system captured. To the authors’ knowledge this is the first application of a vibrometer in an environment as demanding as a powerhouse during operation. By now the technology provides the improvement of signal quality where the estimated normalised cross-correlation value of the corrected signal can reach 0.9 with respect to the control signal, which is a substantial improvement. Original vibrometer amplitudes are overestimated, since the vibrometer movement also increases the amplitude of the relative measurement.

This experiment is the first step in establishing non-contact measurements using a portable vibrometer as a sufficient alternative to traditional measurements inside a hydropower dam. There is still a substantial amount of work to continue this research, to reach the goal when the technology is robust enough to be used during all operational regimes and included in the system of regular structural health monitoring of the dam. Further improvements are possible, especially to tackle the susceptibility to magnetism by applying technological improvements to the design of the DAQ system, EM shielding should be considered and measures to improve EMC immunity applied. The piezoelectric AC accelerometers were used due to their small size however, the frequency response of AC accelerometers cannot be extended down to the DC component. The use of charge amplifiers improves the sensitivity in the low frequency range, it can come close to 1 Hz, while in cases when sensitivity to the DC component is necessary, piezoresistive accelerometers may have to be used. The use of DC accelerometers will improve the the results in the low frequency range. However, the interpretation of signals close to DC should be done with care. Moreover, charge-driven systems have a benefit over voltage-driven systems as they are not sensitive to the length of the connecting cable.

## Figures and Tables

**Table 1:** Power spectrum of turbine operation.

**Frequencies in the power spectrum** [Hz]	1.78, 3.56, 7.15, 21.43, 35.7, 42.85, 85.7, 100
